# Supplementary material for: Free Fatty Acids’ Level and Nutrition in Critically Ill Patients and Association with Outcomes: A Prospective Sub-Study of PermiT Trial
Source: Nutrients. 2019 Feb 13;11(2):384. doi: 10.3390/nu11020384 (PMC6412238; doi:10.3390/nu11020384)
Supplement: Supplementary file 1 [file nutrients-11-00384-s001.pdf]

Table S1: Pearson correlations among baseline free fatty acids (FFAs) level and other related measures of lipid metabolism.

Variables		FFAs	Glucose	LDL	HDL	Cholesterol	Non-HDL cholesterol	Triglyceride	Age	BMI
**LDL**	r	**0.43**	-0.10	1.00	**0.39**	**0.80**	**0.73**	-0.06	0.15	0.11
	P value	**0.0003**	0.43		**0.001**	**<0.0001**	**<0.0001**	0.65	0.21	0.36
**HDL**	r	**0.30**	-0.07	**0.39**	1.00	**0.43**	0.08	**-0.44**	-0.02	-0.19
	P value	**0.01**	0.57	**0.001**		**0.0003**	0.49	**0.0002**	0.90	0.13
**Cholesterol**	r	**0.45**	0.02	**0.80**	**0.43**	1.00	**0.93**	**0.33**	0.20	0.18
	P value	**0.0001**	0.90	**<0.0001**	**0.0003**		**<0.0001**	**0.006**	0.11	0.14
**Non-HDL cholesterol**	r	**0.38**	0.05	**0.73**	0.08	**0.93**	1.00	**0.54**	0.22	**0.27**
	P value	**0.002**	0.72	**<0.0001**	0.49	**<0.0001**		**<0.0001**	0.07	**0.02**
**Triglyceride**	r	0.10	0.04	-0.06	**-0.44**	**0.33**	**0.54**	1.00	-0.01	0.21
	P value	0.41	0.75	0.65	**0.0002**	**0.006**	**<0.0001**		0.93	0.09
**Age**	r	**0.40**	**0.38**	0.15	**-0.02**	0.20	0.22	-0.01	1.00	**0.37**
	P value	**0.0006**	**0.002**	0.21	**0.21**	0.11	0.07	0.93		**0.002**
**BMI**	r	0.20	0.16	0.11	**-0.19**	0.18	**0.27**	0.21	**0.37**	1.00
	P value	0.09	0.22	0.36	**0.13**	0.14	**0.02**	0.09	**0.002**	

HDL: High density lipoproteins; **LDL**: Low density lipoproteins; **BMI**: body mass index; **r=** correlation coefficient

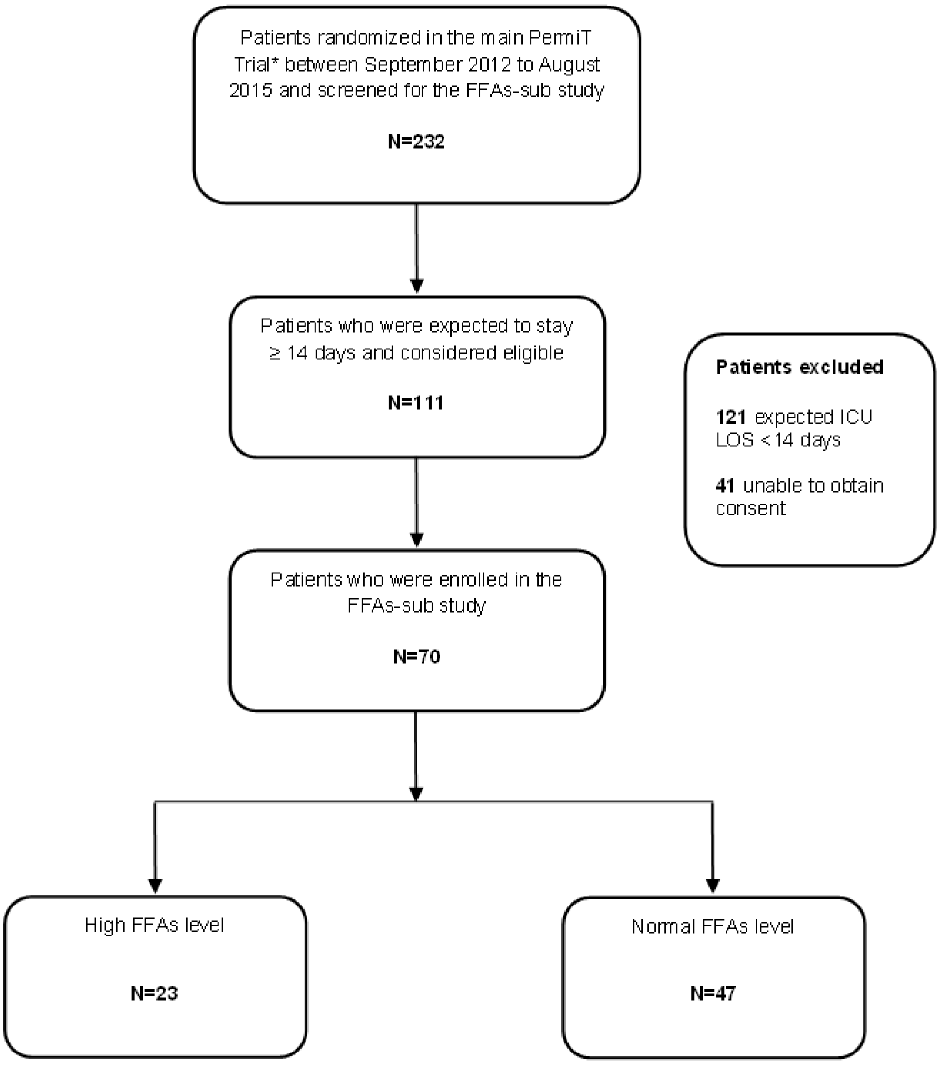

* Arabi YM, Aldawood AS, Haddad SH, Al-Dorzi HM, Tamim HM, Jones G, et al. Permissive Underfeeding or Standard Enteral Feeding in Critically Ill Adults. N Engl J Med. 2015;372(25):2398-408

Figure S1. Flow diagram for patients enrolled in the sub-study of free fatty acids (FFAs) level.
